# Clinical and Epidemiological Profile of Elderly Hodgkin’s Lymphoma in India

**DOI:** 10.7759/cureus.26906

**Published:** 2022-07-15

**Authors:** Manish Sharma, Pankaj Goyal, Rajesh Ranjan, Udip Maheshwari, Dinesh Bhurani, Chaturbhuj Aggarwal, Venkata Pradeep Babu Koyyala, Parveen Jain, Narendra Agrawal, Rayaz Ahmed

**Affiliations:** 1 Medical Oncology, Rajiv Gandhi Cancer Institute and Research Centre, New Delhi, IND; 2 Medical Oncology and Oncology, Rajiv Gandhi Cancer Institute and Research Centre, New Delhi, IND; 3 Community Medicine, Noida International Institute of Medical Sciences, Gautam Budh Nagar, IND; 4 Medical Oncology, Mumbai Oncocare Centre, Mumbai, IND; 5 Hematology and Oncology, Rajiv Gandhi Cancer Institute and Research Centre, New Delhi, IND; 6 Medical Oncology, Bahrain Specialist Hospital, Manama, BHR; 7 Medical Oncology, Assam Cancer Care Foundation, Tezpur, IND; 8 Oncology, Rajiv Gandhi Cancer Institute and Research Centre, New Delhi, IND

**Keywords:** cd positivity, immunohistochemistry, malignancy, elderly, hodgkin’s lymphoma

## Abstract

Background

Hodgkin’s lymphoma (HL) is a curable malignancy that commonly involves the younger population. However, HL can rarely occur in the elderly population (≥60 years) and probably has different biology as compared to the younger counterparts. There was a paucity of data on the clinical and epidemiological profile of the elderly subset with HL in Indian patients who are misdiagnosed and empirically treated as tuberculosis. We have done an analysis of this subset of elderly patients who were registered at our institute.

Methods

A retrospective chart analysis of HL patients who presented to our center from 2008 to 2016 was conducted. Twenty-eight patients with HL of age ≥60 years were included in this study.

Results

Elderly HL comprised 18.67% of the total HL patients registered during this period. The majority were male patients, and the mean age of presentation was 65.9±5.6 years. A Charlson Comorbidity Index (CCI) of ≥2 was seen in 30.77% of the patients. Among these, 84.62% of the patients presented with advanced-stage disease, and 57.69% of the patients presented with B symptoms, which was significantly associated with a high-risk international prognostic score (IPS). Histology-wise, mixed cellularity classical Hodgkin’s lymphoma (MCCHL) and nodular sclerosis classical Hodgkin’s lymphoma (NSCHL) were equally represented (30.76%). Of the patients, 50% had extranodal disease, with the liver being the most frequent site involved. One patient each had bone marrow involvement and bulky disease. CD30, CD15, and CD20 positivity was seen in 84.61%, 50%, and 26.92% of cases, respectively.

Conclusion

Among elderly HL patients, males were more commonly represented than females, and patients more often presented with advanced disease and B symptoms and less often with bulky disease and mediastinal mass. Mixed cellularity classical HL is more common in the elderly subset, and significant comorbidities are present in a higher number of elderly HL patients.

## Introduction

Hodgkin’s lymphoma (HL) is an uncommon malignancy of B-cell origin, and its incidence varies according to age, sex, and geographical areas. Hodgkin’s lymphoma is classified broadly into two types according to the WHO classification: classical Hodgkin’s lymphoma (cHL) and nodular lymphocyte-predominant Hodgkin’s lymphoma (NLPHL). cHL comprises 95% of all HL and is further subclassified into four histological groups: nodular sclerosis (NSCHL), mixed cellularity (MCCHL), lymphocyte-rich (LRCHL), and lymphocyte-depleted (LDCHL) [1]. The characteristic of HL is its age distribution; epidemiological studies describe a bimodal age distribution, first around 30-35 years and the second peak around 55-60 years [2-4]. HL is considered a curable malignancy, and various studies show that this holds true for younger age groups; however, elderly patients fare much worse [5-8]. The incidence of HL in the elderly age group (≥60 years) is around 15%-35% in various studies [9-12]. HL in the elderly is different from the younger subset in various aspects. First, it has been suggested that the disease biology of HL in the elderly is different from that of younger individuals, and MCCHL type is more common in the elderly as opposed to NSCHL [13]. Second, HL is associated with comorbidities in the elderly subgroup and their performance status and tolerance to standard chemotherapy. Third, a similar clinical picture is more commonly seen with infectious diseases, leading to delays in diagnosis and treatment. Finally, the life expectancy of the general population is ever increasing; thus, deciphering the clinico-epidemiological profile of HL in the elderly and future directions to therapy become more important. The aim of this study is to note the clinico-epidemiological profile of elderly lymphoma patients, which has not been reported until now.

## Materials and methods

This was a retrospective analysis of elderly Hodgkin’s lymphoma (eHL) patients registered at the hemato-oncology department of a tertiary care center in North India between 2008 and 2016. During this period, 150 cases of HL were registered and diagnosed, of which 28 patients were of age ≥60 years. All cases were pathologically confirmed by an expert hemato-pathologist. All relevant information was obtained through the hospital-based cancer registry and medical records of the selected patients. Two out of 28 cases were lost to follow-up and therefore were excluded from the analysis. A total of 26 patients were available for analysis. The study was initiated after obtaining IEC approval from Rajiv Gandhi Cancer Institute and Research Centre, New Delhi (letter number: IEC/IRB number RGCIRC/05/076).

Definitions

“B symptoms” were defined as follows: unexplained weight loss of >10% of body weight in the six months preceding the diagnosis, unexplained fever with temperatures >38°C for more than three days, and drenching night sweats. Bulky disease is defined as a mass >10 cm. Advanced disease is defined as Ann Arbor stage IIB, IIIA, IIIB, IVA, and IVB. High-risk patients are defined as patients with an age-adjusted international prognostic score (IPS) of 4-6.

Functional status

The Charlson Comorbidity Index (CCI) was used to assess the functional status, which has been validated in elderly lymphoma patients. Retrospective data was used to calculate the score, and lymphoma as a comorbidity was excluded [14-16]. CCI scores of 0-1 and ≥2 were categorized, and scores of ≥2 were considered significant comorbidity.

## Results

In the present study, elderly HL comprised 18.67% (28/150) of the total HL patients registered from 2008 to 2016. Two out of the 28 elderly HL cases were lost to follow-up and so were excluded from the analysis. A total of 26 patients were available for analysis. In the present study, 73.08% (19/26) were males and 26.92% (7/26) are females, and the mean age was 65.9±5.6 years with a range of 60-80 years (Table 1).

**Table 1:** Age and sex distribution of elderly HL cases (N=26).

Sex/age group	60-65 (year)	>65 (year)	Overall
Male	13 (68.42%)	6 (31.58%)	19 (73.08%)
Female	4 (57.14%)	3 (42.86%)	7 (26.92%)
Overall	17 (65.38%)	9 (34.62%)	26 (100%)

In the present study, 34.62% (9/26) of the patients had two or more comorbidities, and six (66.67%) out of these nine patients with two or more comorbidities were of age >65 years. A CCI score of ≥2 was seen in 30.77% (8/26) of cases. Of the patients, 88.46% (23/26) were of ECOG PS 0 and 1, and only 11.54% (3/26) of the patients have PS 2 or worse (Table 2).

**Table 2:** Distribution of functional status among elderly HL cases (N=26).

Functional status	60-65 (year)	>65 (year)	Overall
Comorbidities			
Two or more comorbidities	6 (66.67%)	3 (33.33%)	9 (34.62%)
0-1 comorbidity	11 (64.71%)	6 (35.29%)	17 (65.38%)
CCI scores			
0-1	14 (77.78%)	4 (22.22%)	18 (69.23%)
≥2	3 (37.50%)	5 (62.50%)	8 (30.77%)
ECOG PS			
0-1	16 (69.57%)	7 (30.43%)	23 (88.46%)
Two or worse	1 (33.33%)	2 (66.67%)	3 (11.54%)

In the present study, the most common presenting symptom seen in patients was neck swelling (65.38%, 17/26), followed by B symptoms (57.69%, 15/26) and abdominal pain (34.61%, 9/26). Of the patients, 15.38% (4/26) presented with early-stage disease, and 84.62% (22/26) of the patients presented with advanced-stage disease. In advanced HL, 59.09% (13/22) of the patients were at high risk by IPS, and 40.91% (9/22) were at low risk by IPS. Of the patients, 73.33% (11/15) with B symptoms were in the high-risk IPS category (Table 3). B symptoms were significantly associated with high-risk IPS patients (p=0.046).

**Table 3:** Clinical presentation of elderly HL cases (N=26).

Clinical presentation	High IPS category (n=15)	Low IPS category (n=11)	p-value
Disease stage			
Early (n=4)	2 (50%)	2 (50%)	0.735
Advanced (n=22)	13 (59.09%)	9 (40.91%)	
B symptoms			
No (n=11)	4 (36.36%)	7 (63.64%)	0.046
Yes (n=15)	11 (73.33%)	4 (26.67%)	

**Figure 1:**
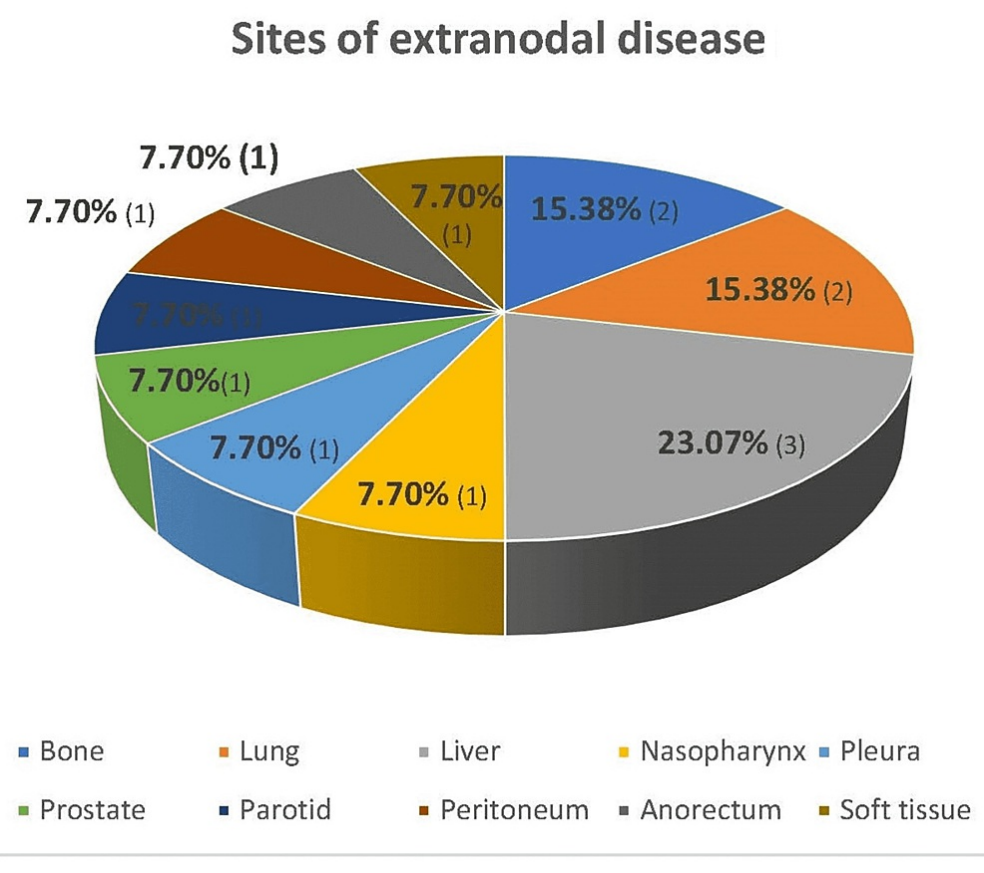
Distribution of extranodal disease sites of elderly HL cases (N=13).

Of the patients, 50% (13/26) presented with extranodal disease, and the most common site was the liver (23.07%, 3/13), followed by bone (15.38%, 2/13) and the lung (15.38%, 2/13) (Figure 1). Bulky disease and bone marrow involvement were seen in one patient each, and no patient had a large mediastinal mass.

**Figure 2:**
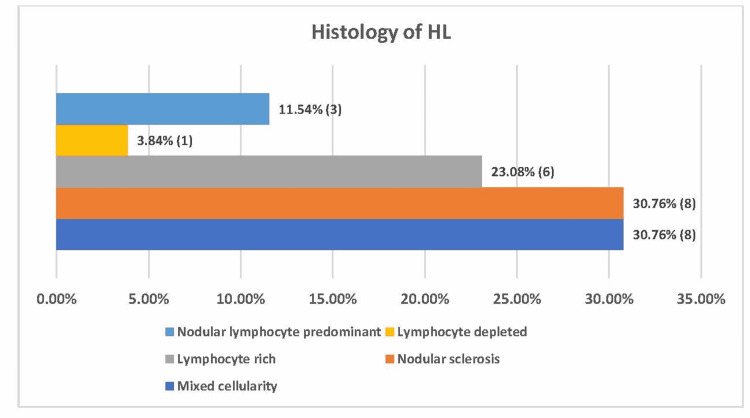
Distribution of Hodgkin’s lymphoma subtypes among the patients (N=26).

**Figure 3:**
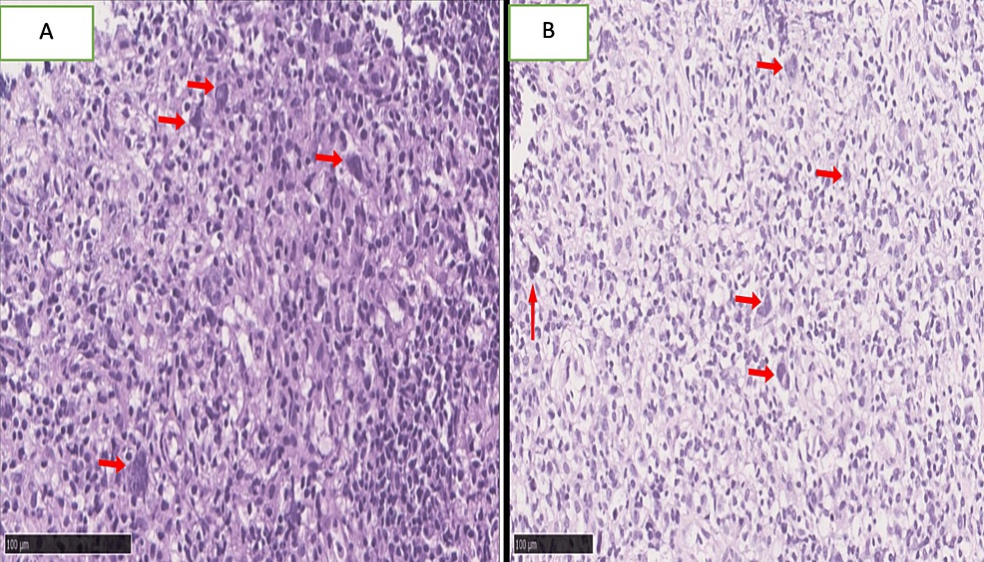
Histological features of nodular lymphocyte-predominant Hodgkin’s lymphoma (NLPHL). (A,B) Hematoxylin and eosin stain at low magnification (×100) shows the vaguely nodular architecture of NLPHL (arrows).

In the present study, the most common type of HL was cHL (88.46%, 23/26), and the remaining were nodular lymphocyte-predominant (11.54%, 3/26). The most common subtypes of cHL observed were MCCHL and NSCHL, with a percentage of 30.76% (8/26) (Figure 2 and Figure 3).

**Figure 4:**
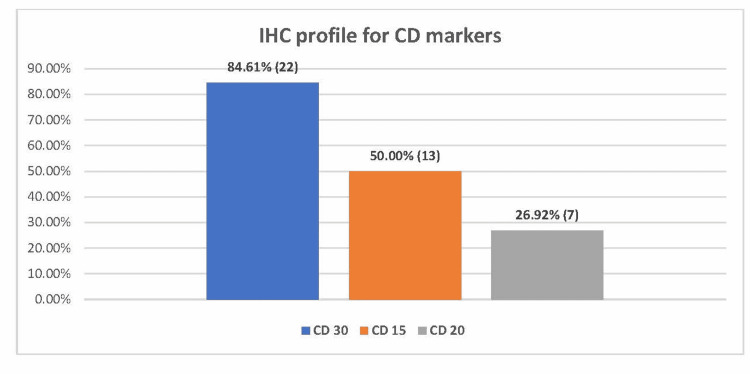
IHC profile for CD markers of Hodgkin’s lymphoma among the patients (N=26).

**Figure 5:**
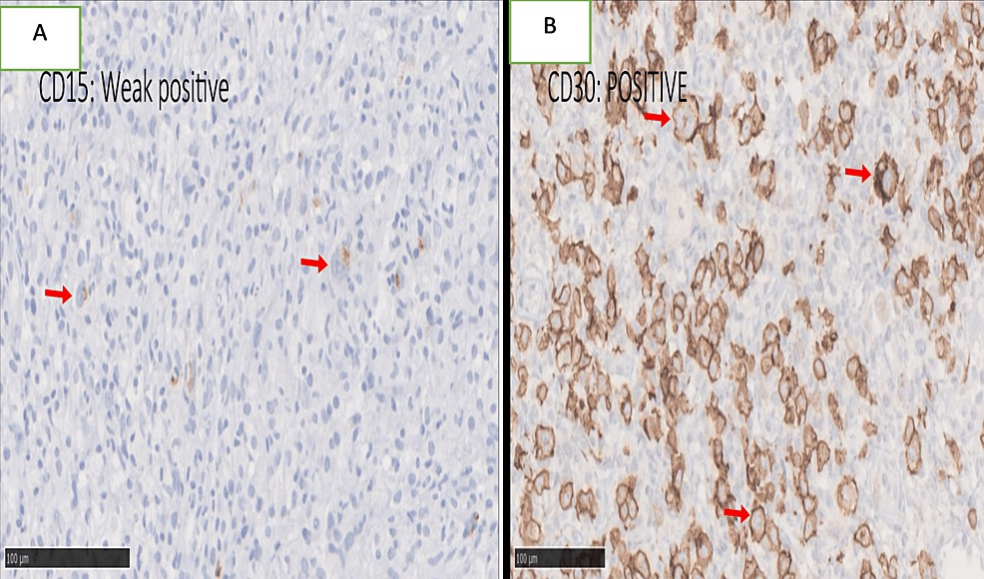
Immunophenotypic features of nodular lymphocyte-predominant Hodgkin’s lymphoma (NLPHL). (A,B) Immunohistochemical studies (×100) show a typical immunophenotype of classical Hodgkin’s lymphoma in the atypical cells with the expression of CD15 (A) and CD30 (B) (arrows).

Immunohistochemistry (IHC) showed that 84.61% (22/26) of the patients were CD30-positive, 50% (13/26) were CD15-positive, and 26.92% (7/26) were CD20-positive (Figure 4 and Figure 5).

## Discussion

Elderly HL behaves differently than HL in younger patients with respect to incidence, sex, prognostic factors, clinical presentation, and histology. Most of the available data is retrospective from the western world. We tried to analyze similar parameters in the Indian population (Table 4).

**Table 4:** Differences in elderly HL and HL in the younger population.

Characteristics	Elderly	Young (<60 years)
Incidence	15%-30% [7,10-12]	70%-80%
Sex (M/F)	2:1 [2,10,17]	1:1 [17]
Comorbidities	50% [15,16]	13% [16]
Bulky disease	Less common [6]	More common
Sub-diaphragmatic presentation	More common [6,16]	Less common
B symptoms	More common [6]	Less common
Mediastinal mass	Less common [11]	More common
ESR	Elevated [11]	Less elevated
EBER/LMP positivity	30%-50% [7,18]	Less common [18]
Mixed cellularity	35%-50% [2,11,19]	19% [11]
Nodular sclerosis	41% [11]	66% [11]
Other prognostic factor	Age > 70 years [2,10]	Age > 45 years

MacMahon et al. distinguished three age periods (0-14 years, 15-34 years, and 50 years and above) and noted that for the 0-14, 15-34, and 50 and above year group, the male/female distributions were 5.7:1, 1:1, and 2:1, respectively [17]. Our study confirms that more number of males (17/26) are usually affected in the elderly subset, which is also shown in two different retrospective analyses [2,10]. Elderly HL accounts for 18.67% of all HL in our study, which corresponds to the proportion (20%) seen in a population-based study [7]. Functional status evaluation of elderly HL patients has been done in selective studies that show up to 50% of patients with significant comorbidity [15], which is slightly on the higher side as compared to that seen in our study (34.62%); however, it is still more than (13%) that seen in younger patients in a population-based study [15]. B symptoms were more common among the elderly in our study, which is also confirmed by various studies [6,11], and these studies have also shown it to be associated with a worse prognosis. The other similar associations seen in our study and confirmed by other analyses are less bulky disease (>10 cm) and more number of patients presenting with advanced disease [6,7,10,20-22].

Histological differences are quite evident in the elderly population. Engert et al., in their study, showed MCCHL to be more commonly seen in elderly patients (35%) as compared with younger patients (19%), and NSCHL is less frequently observed among elderly patients than in younger patients (41% versus 66%). However, this group suggests that nodular sclerosis is still the most common histology in both age groups [11]. The remaining subtypes are represented with the same frequency in elderly and younger patients. However, other studies showed that mixed cellularity is more common [19]. In our study, we have noted the equal occurrence of both mixed cellularity and nodular sclerosis cHL (30.76%).

Advanced-stage HL is more common as compared to early HL in the elderly; however, on risk stratification by age-adjusted IPS, there are no differences seen in high-risk (4-6 factors) and low-risk patients between elderly and younger HL cases [11]. In our study, we have seen a greater number of patients with high-risk IPS as compared to low-risk IPS, and a significant association of high-risk patients was seen with B symptoms (p=0.046).

Tuberculosis is a very common infectious disease in India, and the lack of adequate medical facilities in rural areas leads to the misdiagnosis of conditions such as lymphoma, which has similar clinical (cervical adenopathy and B symptoms) and radiologic features [23]. This leads to a delay in diagnosis and could be a probable cause for advance stage of presentation [24]. Thus, consideration of lymphoma in such a scenario becomes important for early diagnosis and referral in developing countries such as India.

Classical Hodgkin’s lymphoma (cHL) originates from mature B-cells; however, Hodgkin-Reed-Sternberg cells lose their capacity to express B-cell markers on the cell membrane [25]. Benharroch et al. showed that patients aged >45 years have significantly more CD20 positivity (21%) in their study; however, CD20 expression was not prognostic [26]. Our study also showed a similar result of CD20 positivity of 26.92%. CD30, another diagnostic marker, is a surface marker for neoplastic cells of Hodgkin’s lymphoma [27], and CD30 positivity is seen in 80%-100% of cases of cHL and anaplastic large cell lymphoma [28,29], while CD15 is a characteristic marker in cHL and is seen to be positive in 80% of cases. However, it is not specific [30]. Similar results of CD30 positivity (84.6%) were seen in our study; however, CD15 positivity was less (50%) as compared to other studies. No study has compared CD15 positivity in elderly patients; therefore, no conclusions can be derived from this finding [4].

## Conclusions

This retrospective analysis provides an insight into the clinico-epidemiological profile of elderly Hodgkin’s lymphoma patients from one of the northern states in India. We show differences and comparisons in various factors, including clinical presentation, histology, and comorbidities; however, larger studies and molecular analyses are further required to confirm these findings and delineate the pathophysiology behind these differences. We also want to highlight that HL occurs in a significant proportion of the elderly population with B symptoms and cervical lymphadenopathy in India, where tuberculosis forms the first differential diagnosis and patients were started on antituberculosis therapy, resulting in delayed referral to oncology centers, leading to delay in diagnosis and presentation in the advanced stage.
